# Editorial: HeLa Cell Sequela

**DOI:** 10.1534/g3.113.006304

**Published:** 2013-06-01

Free and informed consent of participants in research studies is a cornerstone of biomedical research, but this has not always been the case. Cells derived from tumor tissue of an American woman without her consent more than 60 years ago and named HeLa cells were eventually identified as belonging to Henrietta Lacks, a fact that became widely known in 2010 upon publication of a bestselling book about the woman and her cellular legacy (Skloot 2009). HeLa cells have been broadly disseminated to laboratories around the globe and have been at the forefront of biomedical research for more than five decades. Many key discoveries that have contributed to our understanding of the basis and mechanisms of life and also to significant advances in health care are attributable to studies that employed HeLa cells.

To fulfill our responsibility to disseminate important research results, the editors of *G3: Genes|Genomes|Genetics*—a peer-reviewed, peer-edited, open-access journal of the Genetics Society of America—published early online an article in which the authors described the genome sequence of a HeLa cell line that promises to increase its utility and reveal its limitations for research. Soon after release of that article, the authors withdrew access to the sequence data in response to concerns about genetic privacy that the Lacks family expressed to Rebecca Skloot about their genetic privacy.

Today, regulations in the United States and elsewhere require that consent be obtained before deriving a cell line from an identified person’s tissue. But what should be done with useful cell lines derived long ago without proper consent, or in cases in which that consent could not anticipate future uses? Do we have obligations to the descendants of the original “donor?” Our recently acquired ability to decode DNA sequences of whole genomes has brought this dilemma into sharp focus.

As responsible academic researchers and editors, we must seize this opportunity to address, in a constructive way, the questions raised by the study. In the short term, the editors of *G3* will work with the authors of the study to make the sequence data available in a way that responds to the concerns of the Lacks family.

One thing is clear: the sequence information will add immeasurable value to the large amount of genetic information available in the public domain about the HeLa cell genome, helping ensure that these cells continue to catalyze advances in cell and cancer biology. We must arrive at a resolution soon because publishing an article while withholding access to the underlying (genomic) data is incongruent with our goals as scientists and publishers.

Beyond the short term, the Genetics Society of America intends to encourage thoughtful conversations on the ethical issues raised by this case, with the goal of helping to develop common standards for the acquisition and distribution of personal genomic information. Because of the significance of the questions, those conversations should engage those with appropriate expertise and experience, away from the “madding crowd’s [sometimes] ignoble strife.” But we must move with dispatch to engage the entire community in seeking solutions because everyone—scientists, clinicians, ethicists, and the public—has a stake in the outcome. Please collaborate with us in this important discussion.
